# Changes in human gut flora with age: an Indian familial study

**DOI:** 10.1186/1471-2180-12-222

**Published:** 2012-09-26

**Authors:** Nachiket Marathe, Sudarshan Shetty, Vikram Lanjekar, Dilip Ranade, Yogesh Shouche

**Affiliations:** 1 Microbial Culture Collection, National Centre for Cell Science, NCCS Complex, Ganeshkhind, Pune- 411 007, Maharashtra, India; 2 Agharkar Research Institute, Gopal Ganesh Agarkar Road, Pune – 411004, Maharashtra, India

**Keywords:** Indian population, *Firmicutes/Bacteroidetes* ratio, Human gut microflora, YY-paradox

## Abstract

**Background:**

The gut micro flora plays vital role in health status of the host. The majority of microbes residing in the gut have a profound influence on human physiology and nutrition. Different human ethnic groups vary in genetic makeup as well as the environmental conditions they live in. The gut flora changes with genetic makeup and environmental factors and hence it is necessary to understand the composition of gut flora of different ethnic groups. Indian population is different in physiology from western population (YY paradox) and thus the gut flora in Indian population is likely to differ from the extensively studied gut flora in western population. In this study we have investigated the gut flora of two Indian families, each with three individuals belonging to successive generations and living under the same roof.

**Results:**

Denaturation gradient gel electrophoresis analysis showed age-dependant variation in gut microflora amongst the individuals within a family. Different bacterial genera were dominant in the individual of varying age in clone library analysis. Obligate anaerobes isolated from individuals within a family showed age related differences in isolation pattern, with 27% (6 out of 22) of the isolates being potential novel species based on 16S rRNA gene sequence. In qPCR a consistent decrease in *Firmicutes* number and increase in *Bacteroidetes* number with increasing age was observed in our subjects, this pattern of change in *Firmicutes / Bacteroidetes* ratio with age is different than previously reported in European population.

**Conclusion:**

There is change in gut flora with age amongst the individuals within a family. The isolation of high percent of novel bacterial species and the pattern of change in *Firmicutes /Bacteroidetes* ratio with age suggests that the composition of gut flora in Indian individuals may be different than the western population. Thus, further extensive study is needed to define the gut flora in Indian population.

## Background

The gut micro flora plays an important role in health status of the host as it contributes to overall metabolism and plays a role in converting food into nutrients and energy [1]. Majority of microbes residing in the gut have a profound influence on human physiology and nutrition and are crucial for human life [2-4]. Gut microbiota shapes the host immune responses [5]. The composition and activity of indigenous gut microbiota are of paramount importance in the health of individual and hence describing the complexity of gut flora is important for defining its effect on human health. The limited sensitivity of culture based method has been a problem in the past for defining the extent of microbial diversity in human gut. Recently, the molecular methods used for studying the human gut flora have facilitated the accurate study of the human gut flora. Such studies showed that the human gut microbiota varies greatly with factors such as age, genetic composition, gender, diseased and healthy state of individual. [6-9]. Majority of the gut microbiota is composed of strict anaerobes, which dominate the facultative anaerobes and aerobes by two to three orders of magnitude [10,11]. Although there have been over 50 bacterial phyla described, the human gut microbiota is dominated by only two of them: *Bacteroidetes* and *Firmicutes* while *Proteobacteria*, *Verrucomicrobia*, *Actinobacteria*, *Fusobacteria*, and *Cyanobacteria* are present in minor proportions [12,13]. Studies have shown that the ratio of *Firmicutes* / *Bacteroidetes* changes during challenged physiological conditions such as obesity [14,15], although other studies did not observe any change [16,17]. Changes in *Firmicutes* / *Bacteroidetes* ratio have also been reported in other physiological conditions such as ageing and diabetes [18,19].

Different human ethnic groups vary in genetic makeup as well as the environmental conditions they live in. The gut flora changes with genetic makeup and environmental factors and hence, it is necessary to understand the composition of gut flora of different ethnic groups [20]. However, little effort has been put into understanding the composition of gut flora in Indian population. The physiology of Indian population is different from western population as suggested by YY- paradox and in turn the composition of gut microbes would be different [21]. Hence, in this study we explored the change in composition of gut microbiota in Indian individuals with different age within a family by using culture dependent and molecular techniques. We selected two families each with three individuals belonging to successive generations living under the same roof. Stool samples were collected and DNA extraction, DGGE analysis, preparation of 16S rRNA gene clone libraries was done and the results were validated by qPCR. Obligate anaerobes were isolated from samples collected from one family to study the culturable diversity differences. Our results demonstrate the variation in gut microflora with age among individuals within a family; in addition the pattern of change in *Firmicutes / Bacteroidetes* ratio with age is different to what is previously reported in European population [16].

## Methods

### Selection criteria for subjects and sample collection

Subjects from two healthy Indian joint-middle class families with similar eating habits comprising of three successive generations staying under one roof and with no history of gastrointestinal diseases, no genetic disorders and no antibiotics consumed in the past six months were selected. Age of individuals in Family S was S1 (26 years), S2 (8 months), and S3 (56 years) and in family T was T1 (14 years), T2 (42 years), and T3 (62 years). Stool samples were collected in a sterile N2 flushed bottles on the same day from each individual within a family and within 2 hours were transported to laboratory. Samples of family S were processed for isolation of strict anaerobes and remaining samples from both the families were frozen at −70°C for further molecular analysis. All the experiments were carried out with approval from Institutional (NCCS, Pune) Ethical Committee. A written informed consent was obtained from the subjects, in case of children written consent was obtained from their parents.

### Isolation of strict anaerobes

Three samples from family S were processed for isolation study. Each sample was serially diluted in pre-reduced sterile phosphate buffer (pH 7.0) 0.3 g, K_2_HPO_4_, 0.18 g, KH_2_ PO_4_ , 0.45 g, NaCl, 0.46 g, (NH _4_) 2SO_4_ , 0.05 g, CaCl_2_ , 0.09 g, Mg_2_ SO_4_ ; H_2_O, 0.001 g, resazurin, 0.5 g, L- cysteine HCl; H_2_O and observed under phase contrast microscope (Nikon Eclipse 80*i*, Japan) in order to obtain morphological details and density of bacteria (cells ml^-1^). Serial dilutions were carried and 0.1 ml of each dilution from 10^-5^ to 10^-8^ of the fresh sample were placed on the pre-reduced medium agar plates in an anaerobic chamber (Anaerobic system 1029, Forma Scientific Inc., USA) with gas phase of N_2_:H_2_:CO_2_ (85:10:5). The plates were incubated at 37°C in built-in incubator in the anaerobic chamber. Two non-selective media namely Peptone Yeast Extract Glucose (PYG), Brain Heart Infusion (BHI) (OXOID LTD., England) and one selective medium namely Bile Esculin (BE) were used for the isolation.

Enrichments were set up for all fecal samples in PYG, BHI and BE medium to culture bacteria present in low numbers in the feces. One gram of fecal sample was suspended in 9 ml pre-reduced sterile broth. After consecutive transfers to enrich different bacteria, the enrichment cultures were serially diluted up to 10^-8^. The last four dilutions were placed on the pre-reduced respective medium agar plates under anaerobic conditions and were kept for incubation at 37°C.

Direct isolation and enrichment plates were incubated for 5 days and well grown morphologically different colonies were picked after every 24 h during the 5 days incubation. Transfer of selected colony into the liquid medium was performed in the anaerobic chamber and the purity of the isolates was confirmed by microscopy and re-isolation. The nature of growth (obligate/facultative) was confirmed by growing isolates in pre-reduced PYG medium under both aerobic and anaerobic conditions. Out of 57 isolates obtained only 22 were confirmed as obligate anaerobes and were taken for further studies. Colony morphologies were observed after 3 days of incubation. Cellular morphology was recorded after gram staining of 48 hours old culture. Hanging drop preparation of 24 hour old culture broth was examined under phase contrast microscope for cellular motility [22].

### Extraction of genomic DNA from isolates and community DNA extraction from stool samples

The DNA was extracted from freshly grown cultures using standard Phenol: Chloroform method [23]. Total community DNA was extracted from stool samples using QIAmp DNA Stool Mini kit (Qiagen, Madison USA) following manufacturer’s protocol.

### Identification of isolates by 16S rRNA gene sequence analysis

The isolates were identified by 16S rRNA gene sequencing using universal primer set 27F (5'-CCAGAGTTTGATCGTGGCTCAG-3') and 1488R (5'-CGGTTACCTTGTTACGACTTCACC-3') [24]. All the PCR reactions were carried out in a total volume of 25 μl. The reaction constituted 1X standard Taq Buffer, 200 nM dNTPs, 0.4 μM of each primers , 0.625 U Taq Polymerase (Banglore Genei, Banglore India) and 20 ng of template DNA. All PCR were performed for 35 cycles. Purified PCR products were sequenced using BigDye Terminator Cycle Sequencing Ready Reaction Kit v 3.1 in an automated 3730xl DNA analyzer (Applied Biosystems Inc, USA).

### Biochemical characterization of the isolates

Biochemical characterization of the isolates was done using BIOLOG AN microplate following BIOLOG^TM^ assay [25] and identified according to Bergey’s Manual for Systematic Bacteriology. The pure cultures of anaerobic bacteria grown on petri plates in anaerobic chamber (Forma Scientific, USA) were inoculated in Biolog anaerobic inoculating fluid and the turbidity of the inoculum was adjusted according to Biolog protocol. Hundred micro liter of the inoculum was pipetted into each well of 96 well AN microplates and incubated at 37°C in in-built incubator in anaerobic chamber. Incubation period varied from 48 to 72 hrs depending on the growth of the bacteria.

### DGGE analysis of the community DNA

The Denaturation Gradient Gel Electrophoresis (DGGE) PCR was done for the community DNA using the primers 358F (40 GC 5’-CTACGGGAGGCAGCAG-3’) and 517R (5’-CCGTCAATTC(A/C)TTTGAGTTT -3’) modified linker primers [26]. The DGGE was performed in 10% acrylamide: bis acrylamide (37.5:1) gel with a gradient of 40% to 60%. One hundred percent of the denaturant corresponds to 7 M urea and 40% deionized formamide. The electrophoresis was done using DCode Universal Mutation Detection System (BioRad, Hercules, CA, USA) at 80 V for 18 h at 60^0^ C. The gel was run in 1 X TAE buffer (40 mM Tris, 20 mM Sodium acetate, 1 mM EDTA) and stained with ethidium bromide. The documentation of gel was done using Syngene G: box gel documentation system (Syngene, Cambridge, UK).

### Clone library preparation from community DNA

Total community DNA was used for preparing 16S rRNA gene libraries. The 16S rRNA gene was amplified with modified universal primers for bacteria 8FI (5’GGATCCAGACTTTGATYMTGGCTCAI-3’) and 907RI (5’- CCGTCAATTCMTTTGAGTTI-3’) [27]. The PCR product were purified by gel elution using Gene Elute Gel Extraction Kit (Sigma-aldrich, St Louis USA) and were ligated into pCR4® TOPO vector supplied with the TOPO TA cloning kit (Invitrogen, San Diego, USA) and transformed into One Shot TOPO10 electrocompetent cells of *E. coli* (Invitrogen, San Diego, USA) following the manufacturer’s instructions. Sterile LB agar with 50 μg/ml of kanamycin were used for selection of the transformed cells which were incubated for 16 h at 37°C. M13F and M13R primers were used for screening and sequencing of the clones. The sequencing was done by ABI 3730 XL DNA analyser (Applied Biosystems Inc, USA) using the ABI Big-Dye terminator version 3.1 sequencing kit as per the manufacturer’s instructions.

### Phylogenetic analysis

Sequences from each of the clone libraries were compared to the current database of 16S RNA gene sequences at Ribosomal Database Project II [28]. The sequences were assembled and contig’s were obtained using ChromasPro software, alignment was done using CLUSTAL X2 and the sequences were edited manually using DAMBE to get unambiguous sequence alignment. All sequences were checked for chimeric artifacts by Mallard program, reference sequence used for this purpose was *E. coli* U000096 [29] Appropriate subsets of 16S rRNA gene sequences were selected on the basis of initial results and subjected to further phylogenetic analysis using DNADIST of Phylip (version 3.61). The number of Operational Taxonomic Units (OTU) (clone sequences with > 97% similarity grouped together as one OTU) were obtained by DOTUR program (version 1.53) using furthest neighbor algorithm [30]. Representative sequences from each of the OTUs were retrieved and checked against the previously determined 16S rRNA gene from the RDPII release 10 version of the database and these sequences were downloaded in FASTA format. Phylogenetic analyses were conducted using *MEGA,* version 4 [31], and the phylogenetic trees were constructed using neighbor-joining method with Kimura 2 parameter [32,33]. Normalized heat map was generated using MG-RAST, a modified version of RAST server, using RDP database [34].

### Real time PCR

The Real Time PCR was done using the 7300 Real time PCR system from Applied Biosystems Inc. (USA) using SYBR green master mix (Applied Biosystems Inc. USA). Primers used for absolute quantification were reported earlier [19]. The primers used are listed in Table 1.

**Table 1:** Primers used for Real-Time PCR

**Target organism**	**Primer**	**Sequence**	**PCR product (bp)**
*Clostridium coccoides-Eubacteria rectale* group	**ClEubF**	CGGTACCTGACTAAGAAGC	429 [47]
	**ClEubR**	AGTTTYATTCTTGCGAACG	
*Prevotella*	**PrevF**	CACCAAGGCGACGATCA	283 [19]
	**PrevR**	GGATAACGCCYGGACCT	
*Lactobacillus* group	**LacF**	AGCAGTAGGGAATCTTCC	341 [48]
	**LacR**	ACACCGCTACACATGGAG	
*Bacteroides-Prevotella* group	**BacF**	GAAGGTCCCCCACATTG	410 [49]
	**BacR**	CAATCGGAGTTCTTCGTG	
*Bifidobacterium*	**BifF**	GCGTGCTTAACACATGCAAGTC	126 [50]
	**BifR**	CACCCGTTTCCAGGAGCTATT	
*Roseburia*	**RosF**	TACTGCATTGGAAACTGTCG	230 [19]
	**RosR**	CGGCACCGAAGAGCAAT	
All bacteria	**27F**	TCCTACGGGAGGCAGCAGT	316 [This study]
	**343R**	GACTACCAGGGTATCTAATCCTGTT	

Legend: ClEub*- Clostridium coccoides-Eubacteria rectale* group specific primers, *Prev- Prevotella genus* specific primers, *Lac- Lactobacillus genus* specific primers, *Bac-Prev- Bacteriodes-Prevotella* specific primers, *Bif- Bifidobacterium genus* specific primers *, Ros- Roseburia genus* specific primers and *All bacteria-* universal primers for all bacteria.

Standards were prepared using these primers and the PCR products were gel eluted using Gene Elute Gel Extraction Kit (Sigma-aldrich, St Louis USA). The gel eluted products were quantitated using nanodrop ND-1000 spectrophotometer (JH Bio innovations, Hyderabad India) and serial dilutions were made as standards. Efficiency of PCR was calculated using the equation E = 10^-1/slope^ – 1 where, E is efficiency of PCR, mass of genome was calculated using the equation M = (n) - 1.096e-21 g/bp where M is mass of genome and n is the PCR product size. The normalization was done by dividing the copy numbers of each bacterial genus with total bacteria copy number. The *Firmicutes* /*Bacteroidetes* ratio was calculated by dividing the normalized copy numbers of *Lactobacillus* group + *Clostridium coccoides-Eubacteria rectale* group by the copy number of *Bacteroides-Prevotella* group [18].

## Results

### Biochemical and molecular characteristics of the human fecal isolates

Total 22 strict anaerobic bacteria isolates were obtained from human fecal samples from three healthy volunteers. These bacterial isolates were identified using 16S rRNA gene sequence analysis. Different bacterial species were isolated from different aged individuals with infant showing the least diversity (only two species were isolated) with 4 isolates being *Parabacteroides distasonis* and 1 isolate being *Bifidobacterium adolscentis.* The isolates from samples S1 and S3 belonged to genus *Bacteriodes*, *Clostridium, Parabacteroides*; while *Megasphaera elsdenii* was isolated from S3 only (age56)*.*This suggests that there is difference in culturable anaerobic bacteria diversity with age within individuals in a family.

None of the isolate showed 100% sequence similarity with the known sequences in database, with 27% (6 out of 22) of the isolates showing 97% or less similarity to the type strains suggesting that they are novel species. These potential novel isolates were closely related to 6 different bacterial species belonging to 5 different genera (Table 2), suggesting a high diversity of novel bacterial species. The isolation of novel species also showed age related difference among the individuals, novel species closely related to *Bifidobacteria adolescentis* was isolated only from infant while novel species closely related to *Clostridium difficile* was isolated only from S1 (adult). The sample S3 showed high diversity of novel isolates with presence of 4 novel isolates closely related to *Parabacteroides distasonis*, *Megasphaera elsdenii*, *Clostridium subterminale*, *Bacteroides fragilis* respectively. This suggests that there is difference in culturable anaerobic bacteria diversity with age within individuals in a family.

**Table 2:** Identification of obligate anaerobic isolates by 16 S rRNA gene sequence analysis

**Sample**	**Isolate**	**Closest BLAST hit**	**Percent similarity**	**Gene bank accession numbers**
S2	SLPYG 1	*Bifidobacteria adolescentis*	97%	JN389522
(8 months)	SLPYG 2	*Parabacteroides distasonis*	99%	JN038555
	SLPYG 3	*Parabacteroides distasonis*	99%	JN038556
	SLBE 4	*Parabacteroides distasonis*	99%	JN038557
	SLBE 5	*Parabacteroides distasonis*	99%	JN038558
S1	VLPYG 2	*Clostridium subterminale*	99%	JN093125
(26 years)	VLPYG 3	*Bacteroides vulgates*	99%	JN084207
	VLPYG 4	*Parabacteroides distasonis*	99%	JN038554
	VLPYG 5	*Clostridium difficile*	96%	JN093126
	VLPYG 6	*Clostridium mangenotii*	98%	JN093127
	VLBE 7	*Bacteroides fragilis*	99%	JN084198
	VLBE 8	*Bacteroides thetaiotaomicron*	99%	JN084201
	VLBE 9	*Bacteroides thetaiotaomicron*	99%	JN084202
S3	BLBE 1	*Parabacteroides distasonis*	97%	JN038559
(56 years)	BLBE 2	*Bacteroides ovatus*	98%	JN084211
	BLPYG 5	*Bacteroides uniformis*	99%	JN084205
	BLBE 6	*Bacteroides xylanisolvens*	99%	JN084212
	BLPYG 7	*Megasphaera elsdenii*	97%	HM990964
	BLPYG 8	*Clostridium subterminale*	96%	JN093128
	BLPYG 9	*Bacteroides fragilis*	97%	JN084199
	BLBE 11	*Parabacteroides distasonis*	99%	JN038560
	BLBE 12	*Parabacteroides distasonis*	99%	JN038561

Biochemical characteristics of the isolates were analyzed using BIOLOG^TM^. The isolates were grouped in 5 different phenotypes based on obtained characteristics. The identifications and accession numbers of the 16SrRNA gene sequence of the isolates are represented in Table 2.

### DGGE analysis

**Figure 1:**
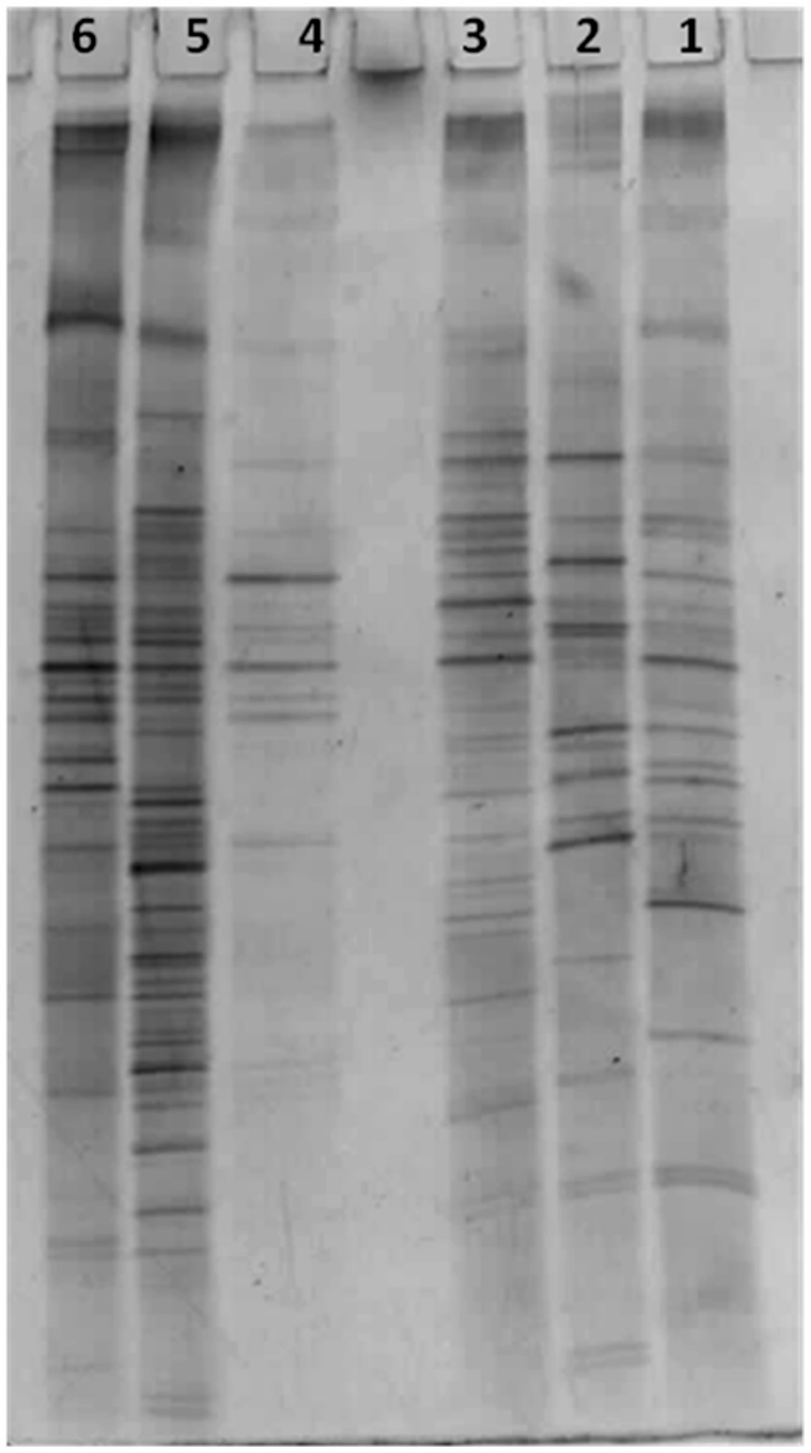
**DGGE analysis of the stool DNA, denaturation gradient 40%-60%. **Family S: S1 (26 years), S2 (8 months), S3 (56 years) and Family T: T1 (14 years), T2 (42 years), T3 (62 years). Legend : Lane 1- S2, lane 2- S1, lane 3- S3, lane 4- T1, lane 5- T2, lane 6- T3.

The DGGE analysis revealed the difference in gut flora composition of individuals of different age belonging to the same family as shown in Figure 1. The band intensity and number of bands observed in DGGE profile of samples suggests that different bacterial species are dominating the gut flora of individuals of varying age.

### Clone library analysis

**Figure 2:**
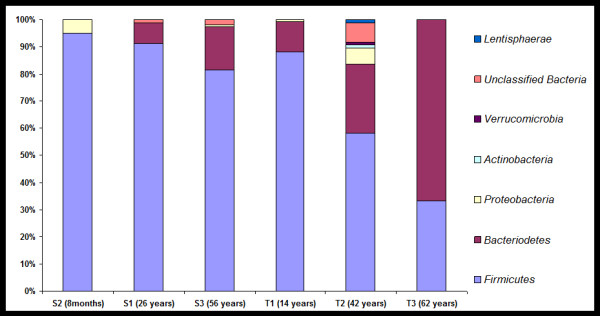
**Phylum level comparison of gut flora of the subjects.** The stacked bars describe the percent distribution of each phylum across the subjects.

**Figure 3:**
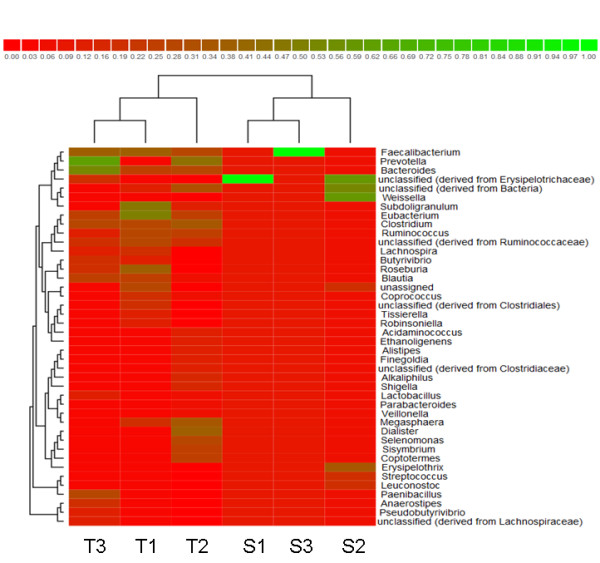
**Genus level comparison of gut flora.** The heat map represents clustering of bacterial communities across the subjects at the genus level. Family S: S1 (26 years), S2 (8 months), S3 (56 years) and Family T: T1 (14 years), T2 (42 years), T3 (62 years).

Total 960 clone sequences from the 6 clone libraries were obtained and analyzed. The sequences are submitted to NCBI with accession numbers from JQ264784 to JQ265743. On the basis of sequence similarities as obtained from Ribosomal Database Project II (RDP II), the sequences were grouped into Phylum *Firmicutes, Bacteroidetes*, *Proteobacteria, Actinobacteria*, *Verrucomicrobia.* The clone library analysis showed consistent decrease in the *Firmicutes* and consistent increase in *Bacteroidetes* in both the families with an increase in age (Figure 2). The family level variation in microflora in individuals is shown in Additional file 1: Table S1. The genera which were dominant in the individual samples are represented in Figure 3. The heat map represented in Figure 3 shows that the individuals within a same family cluster together when genus level distribution of gut flora is considered. Within family T, *Fecalibacterium* and *Roseburia* dominated in subject T1 (age 14) *Dialister, Prevotella* dominated in subject T2 (age 42) and *Prevotella* in subject T3 (age 62). Within family S the genus *Streptococcus* and *Weissella* dominated in the infant and *Fecalibacterium* and *Roseburia* dominated in adult subjects (age 26 and 62 years respectively). The phylogenetic tree of the OTU’s obtained from all the subjects are represented in Additional files 2: Figures S1, Additional file 3: Figures S2, Additional file 4: Figure S3, Additional file 5: Figure S4, Additional file 6: Figure S5, Additional file 7: Figure S6. The phylogenetic trees consist of clades representing the presence of potential novel bacterial species in the gut flora of the subjects.

### Real time PCR

**Figure 4:**
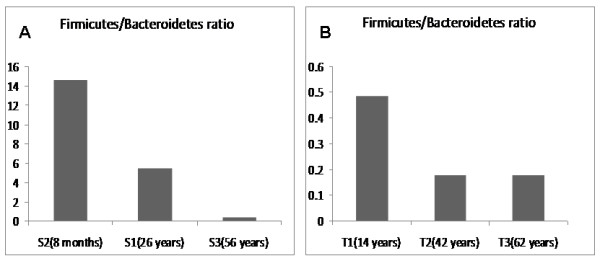
***Firmicutes *to *Bacteroidetes *ratio by qPCR, A- The pattern of change in *Firmicutes/ Bacteroidetes *in family S and B- The pattern of change in *Firmicutes/ Bacteroidetes *in family T.**

The slopes for the standards for all the genus specific primers were in the range of −3.1019 to −3.460 with the R2 value >0.99. The PCR efficiency ranged from 96% to 106%. The qPCR quantification confirmed that the *Firmicutes* number is decreasing and *Bacteroidetes* number is increasing with increasing age. The pattern of change in *Firmicutes*/*Bacteroidetes* ratio with age within a Family is represented in Figure 4. The copy numbers of different genera are represented in Table 3. The copy number of *Roseburia* was more than *Clostridium* and *Lactobacillus* group, suggesting dominance of *Roseburia* in the gut flora, which is consistent with the report by Arumugam *et al.* showing that *Fecalibacterium* and *Roseburia* are the dominant genera in the gut flora [35].

**Table 3:** Copy numbers of different genera in the gut flora of individual samples

**Subjects**	**S2 (8 months)**	**S1 (26 yrs)**	**S3 (56 yrs)**	**T1 (14 yrs)**	**T2 (42 yrs)**	**T3 (62 yrs)**
*ClEub*	2.17 ± 0.9 E + 07	1.91 ± 0.01E + 08	7.85 ± 0.06E + 03	1.08 ± 0.01E + 09	2.19 ± 0.1E + 08	1.17 ± 0.01E + 08
*Prev*	7.83 ± 0.9 E + 07	3.55 ± 0.4E + 07	1.12 ± 0.3E + 08	5.29 ± 0.01E + 07	3.87 ± 0.04E + 08	1.72 ± 0.09E + 10
*Lac*	5.29 ± 0.6 E + 10	3.98 ± 0.5E + 10	3.88 ± 0.5E + 09	3.87 ± 0.3E + 10	1.64 ± 0.2E + 09	1.03 ± 0.5E + 11
*Bac-Prev*	3.61 ± 1.3 E + 09	7.32 ± 0.4E + 09	1.04 ± 0.34E + 10	8.04 ± 0.43E + 10	9.32 ± 0.82E + 10	5.55 ± 0.46E + 11
*Bif*	5.42 ± 0.11E + 07	4.37 ± 0.4E + 08	4.37 ± 0.17E + 06	2.56 ± 0.12E06	2.06 ± 0.6E + 07	1.27 ± 0.5E + 08
*Ros*	1.51 ± 0.26E + 10	1.56 ± 0.2E + 10	3.42 ± 0.19E + 10	2.78 ± 0.15E + 10	1.16 ± 0.40E + 10	1.87 ± 0.54E + 11
*All bacteria*	3.8 ± 0.1E + 10	3.57 ± 0.08E + 10	5.97 ± 0.15E + 10	4.7 ± 0.2E + 11	5.11 ± 0.04E + 11	9.84 ± 0.03E + 11

Legend: ClEub*- Clostridium coccoides-Eubacteria rectale* group specific primers, *Prev-Prevotella genus* specific primers, *Lac- Lactobacillus genus* specific primers, *Bac-Prev-Bacteriodes-Prevotella* specific primers, *Bif- Bifidobacterium genus* specific primers*, Ros-Roseburia genus* specific primers and *All bacteria-* universal primers for all bacteria.

## Discussion

The importance of gut flora in health status and metabolism of the host has been well documented in previous studies [3,4,15]. The development of gut flora is defined by genetics and environmental factors which shape the composition of gut flora in a reproducible manner [20]. In a population as diverse as India, with various ethnic groups living in different geographical areas and having different dietary habits, it is expected that these factors would have an effect on the composition of gut microflora. The differences in composition of gut microflora will in turn have an effect on the host. Hence, it is important to focus on exploring the gut microflora in Indian population. There have been very little reports on Indian gut flora, Pandey *et al.* focused on micro eukaryotic diversity in infants and Balamuragan *et al.* study focused on anaerobic commensals in children and Bifidobacteria in infants [36-38]. We took this opportunity to explore the changes in gut microflora with age within a family. Selecting 3 individuals from the same family means that there is less genetic variation amongst the subjects as compared to non related individuals. A few studies have shown that kinship seems to be involved in determining the composition of the gut microbiota [14,39] and thus selecting related individuals would mean less inter-individual variation in gut flora as compared to unrelated individuals. The subjects are staying in the same house so the variation in the living environmental conditions and feeding habits are lower as compared to individuals staying at different places. Thus, the differences in gut flora observed in this study would be better attributed to changing age. Our results demonstrate that the gut microflora does change within genetically related individuals of different age, living under the same roof. To the best of our knowledge this is the first study focusing on the change in gut flora within a family in Indian population. DGGE analysis (Figure 1) showed that different bacterial species dominate the gut flora in different aged individuals within a family; this finding is consistent with the earlier reports [6,7]. The clone library analysis showed that *Firmicutes* and *Bacteroidetes* are the dominant phyla present in human gut flora in our subjects and also confirmed the results of DGGE analysis showing that different bacterial genera are dominating the gut flora in different aged individuals as shown in Figure 3. The clone library analysis with Sanger sequencing has limitations of having low depth of sequencing as compared to Next generation sequencing technologies like pyrosequencing, however longer read length obtained by Sanger sequencing are beneficial when mapping the sequence to the species level [40]. Fewer than 100 sequences are enough to detect the pattern of variation among the microbial communities in gut of diverse hosts [40-42]. Although clone library analysis would not yield total bacterial diversity, it would give the variation in major bacterial groups within the samples. Recently Zupancic *et al.* reported bacterial genera which forms the core gut microbiota of Amish subjects [43]. We retrieved the sequences for almost all the genera defined as core microbiota by Zupancic *et al.* in our study. This further supports the fact that clone library analysis could be useful in determining the variation in major bacterial phyla in a sample.

A study by Mariat *et al.* on European Population showed that the *Firmicutes* /*Bacteroidetes* ratio being 0.4 in Infants which increases to 10.9 in adults and decreases to 0.6 in elderly [16]. Somewhat different results were observed by Biagi *et al.* in Italian population, the *Firmicutes* /*Bacteroidetes* ratio for adults 3.9 which increased to 5.1 for elderly and decreased to 3.6 for centenarians respectively [44]. Moving from young to elderly the *Firmicutes* /*Bacteroidetes ratio* was observed to be decreased in Mariat *et al.* study while it increased in Biagi *et al.* study [16,44]. In contrast, in our study we observed a consistent decrease in *Firmicutes* number and increase in *Bacteroidetes* number with increasing age. This was observed in the clone library analysis and then validated by qPCR. The decrease in *Firmicutes* number and increase in *Bacteroidetes* suggest that there would be a gradual decrease in *Firmicutes* /*Bacteroidetes* ratio in our subjects with increasing age which further implies that our subjects do not follow the same trend of change in *Firmicutes* /*Bacteroidetes* ratio with age as to what has been reported earlier in European population.

Isolation of strict anaerobes from one of the family showed age related differences in the culturable anaerobic diversity. To the best of our knowledge this is the first study focusing on age related changes in culturable anaerobic diversity from Indian subcontinent. The isolation of *Bifidobacterium adolscentis* from infant sample is consistent with the earlier findings that gut flora is dominated by facultative anaerobes in infants as compared to adult gut flora and *Bifidobacterium* is one of early anaerobic colonizers of infant gut [45,46]. The isolation of highly diverse novel bacterial species from human gut of Indian individuals with varying age suggests Indian population is a good source to find novel bacterial isolates, and might have a different composition compared to the Western Population studied earlier.

This is a preliminary study which investigates a very unique subset of the human gut microflora where 3 generations of a family are living under the same roof. Although the number of families participating in the study is low, the observations of the study are important in context of human gut flora studies in Indian scenario. Much more in-depth study is required to define the gut flora in Indian population; however this study is the stepping stone towards establishment of the changes in gut microflora with age in Indian population.

## Conclusion

The observations of this study suggest that the gut flora of individuals change with age within a family. The Indian population is different in physiology to the western population and our results demonstrate that the gut flora in Indian subjects may be different in composition as compared to the western population [18]. The pattern of change in *Firmicutes*/*Bacteroidetes* ratio with age in our subjects is different from the previously reported pattern in European population. Moreover, the isolation of novel bacterial species demonstrates the fact that human gut flora in Indian population is an unexplored source of potential novel bacterial species. Thus, more effort should be made to extensively define gut flora in Indian population.

## Competing interests

The authors declare that they have no competing interests.

## Authors' contributions

NM and SS were involved in Clone library construction, Phylogenetic analysis, DGGE, qPCR analysis and preparation of manuscript. NM was also involved in identification of the isolates. VL did the isolations of anaerobic bacteria and BIOLOG^TM^ assay. YS and DR designed the study and gave important inputs for preparation of manuscript. All authors have read and approved the manuscript.

## Supplementary Material

Additional file 1 **Table S1.Distribution of different bacterial families in all subjects.** (−) indicates no detection. Click here for file

Additional file 2 **Figure S1.Phylogenetic tree showing the position of 16S rDNA OTU’s recovered from stool sample of S1 individual was constructed using neighbor-joining method based on partial 16S rDNA sequences.** The bootstrap values (expressed as percentages of 1000 replications) are shown at branch points. The scale bar represents genetic distance (2 substitutions per 100 nucleotides). GenBank accession numbers are in parentheses. Click here for file

Additional file 3 **Figure S2.Phylogenetic tree showing the position of 16S rDNA OTU’s recovered from stool sample of S2 individual was constructed using neighbor-joining method based on partial 16S rDNA sequences.** The bootstrap values (expressed as percentages of 1000 replications) are shown at branch points. The scale bar represents genetic distance (2 substitutions per 100 nucleotides). GenBank accession numbers are in parentheses. Click here for file

Additional file 4 **Figure S3.Phylogenetic tree showing the position of 16S rDNA OTU’s recovered from stool sample of S3 individual was constructed using neighbor-joining method based on partial 16S rDNA sequences.** The bootstrap values (expressed as percentages of 1000 replications) are shown at branch points. The scale bar represents genetic distance (2 substitutions per 100 nucleotides). GenBank accession numbers are in parentheses. Click here for file

Additional file 5 **Figure S4.Phylogenetic tree showing the position of 16S rDNA OTU’s recovered from stool sample of T1 individual was constructed using neighbor-joining method based on partial 16S rDNA sequences.** The bootstrap values (expressed as percentages of 1000 replications) are shown at branch points. The scale bar represents genetic distance (2 substitutions per 100 nucleotides). GenBank accession numbers are in parentheses. Click here for file

Additional file 6 **Figure S5.Phylogenetic tree showing the position of 16S rDNA OTU’s recovered from stool sample of T2 individual was constructed using neighbor-joining method based on partial 16S rDNA sequences.** The bootstrap values (expressed as percentages of 1000 replications) are shown at branch points. The scale bar represents genetic distance (5 substitutions per 100 nucleotides). GenBank accession numbers are in parentheses. Click here for file

Additional file 7 **Figure S6. Phylogenetic tree showing the position of 16S rDNA OTU’s recovered from stool sample of T3 individual was constructed using neighbor-joining method based on partial 16S rDNA sequences.** The bootstrap values (expressed as percentages of 1000 replications) are shown at branch points. The scale bar represents genetic distance (5 substitutions per 100 nucleotides). GenBank accession numbers are in parentheses. Click here for file
