# Supplementary material for: Changes in human gut flora with age: an Indian familial study
Source: BMC Microbiol. 2012 Sep 26;12:222. doi: 10.1186/1471-2180-12-222 (PMC3511239; doi:10.1186/1471-2180-12-222)
Supplement: Additional file 1 — Table S1.Distribution of different bacterial families in all subjects. (−) indicates no detection. [file 1471-2180-12-222-S1.doc]

**Supplementary Table S1**: Distribution of different bacterial families in all subjects.

| Subjects  Families | **S2 (8 months)** | **S1 (26yrs)** | **S3 (56yrs)** | **T1 (14yrs)** | **T2 (42yrs)** | **T3 (62 yrs)** |
| --- | --- | --- | --- | --- | --- | --- |
| [Enterococcaceae](http://rdp.cme.msu.edu/seqmatch/seqmatch_sum.jsp?qvector=204&depth=0&currentRoot=0&num=20) | 3.70% | -* | - | - | - | - |
| [Leuconostocaceae](http://rdp.cme.msu.edu/seqmatch/seqmatch_sum.jsp?qvector=204&depth=0&currentRoot=0&num=20) | 42.59% | - | - | - | - | - |
| [Streptococcaceae](http://rdp.cme.msu.edu/seqmatch/seqmatch_sum.jsp?qvector=204&depth=0&currentRoot=0&num=20) | 22.22% | - | - | 0.73% | - | 1.08% |
| [Erysipelotrichaceae](http://rdp.cme.msu.edu/seqmatch/seqmatch_sum.jsp?qvector=204&depth=0&currentRoot=0&num=20) | 25.92% | 12.86% | 2.59% | - | - | 1.08% |
| [Lactobacillaceae](http://rdp.cme.msu.edu/seqmatch/seqmatch_sum.jsp?qvector=204&depth=0&currentRoot=0&num=20) | - | 7.01% | 0.43% | - | 0.59% | 5.43% |
| [Streptococcaceae](http://rdp.cme.msu.edu/seqmatch/seqmatch_sum.jsp?qvector=204&depth=0&currentRoot=0&num=20) | - | 6.43% | 0.43% | - | - | - |
| [Veillonellaceae](http://rdp.cme.msu.edu/seqmatch/seqmatch_sum.jsp?qvector=204&depth=0&currentRoot=0&num=20) | - | 0.58% | - | 1.47% | 23.66% | 3.26% |
| [Ruminococcaceae](http://rdp.cme.msu.edu/seqmatch/seqmatch_sum.jsp?qvector=204&depth=0&currentRoot=0&num=20) | - | 48.53% | 46.75% | 41.17% | 30.17% | 9.23% |
| [Clostridiaceae](http://rdp.cme.msu.edu/seqmatch/seqmatch_sum.jsp?qvector=204&depth=0&currentRoot=0&num=20) | - | 1.16% | - | - | 2.36% | - |
| [Peptostreptococcaceae](http://rdp.cme.msu.edu/seqmatch/seqmatch_sum.jsp?qvector=204&depth=0&currentRoot=0&num=20) | - | 5.26% | - | - | - | - |
| [Incertae Sedis XIV](http://rdp.cme.msu.edu/seqmatch/seqmatch_sum.jsp?qvector=204&depth=0&currentRoot=0&num=20) | - | 1.75% | 3.03% | 8.08% | - | 2.17% |
| [Lachnospiraceae](http://rdp.cme.msu.edu/seqmatch/seqmatch_sum.jsp?qvector=204&depth=0&currentRoot=0&num=20) | - | 8.18% | 21.21% | 33.08% | 6.50% | 8.69% |
| [Eubacteriaceae](http://rdp.cme.msu.edu/seqmatch/seqmatch_sum.jsp?qvector=204&depth=0&currentRoot=0&num=20) | - | 0.58% | - | - | - | - |
| [Unclassified Ruminococcaceae](http://rdp.cme.msu.edu/seqmatch/seqmatch_sum.jsp?qvector=204&depth=0&currentRoot=0&num=20) | - | - | 8.65% | - | - | - |
| [Unclassified Lachnospiraceae](http://rdp.cme.msu.edu/seqmatch/seqmatch_sum.jsp?qvector=204&depth=0&currentRoot=0&num=20) | - | - | 2.16% | 2.94% | - | 2.71% |
| [Enterobacteriaceae](http://rdp.cme.msu.edu/seqmatch/seqmatch_sum.jsp?qvector=204&depth=0&currentRoot=0&num=20) | 5.55% | - | - | - | 5.32% | - |
| [Alcaligenaceae](http://rdp.cme.msu.edu/seqmatch/seqmatch_sum.jsp?qvector=204&depth=0&currentRoot=0&num=20) | - | - | - | 0.73% | - | - |
| [Bacteroidaceae](http://rdp.cme.msu.edu/seqmatch/seqmatch_sum.jsp?qvector=204&depth=0&currentRoot=0&num=20) | - | 4.09% | 4.76% | 11.02% | 6.50% | 3.26% |
| [Rikenellaceae](http://rdp.cme.msu.edu/seqmatch/seqmatch_sum.jsp?qvector=204&depth=0&currentRoot=0&num=20) | - | 1.75% | 7.35% | - | 1.18% | - |
| [Flavobacteriaceae](http://rdp.cme.msu.edu/seqmatch/seqmatch_sum.jsp?qvector=204&depth=0&currentRoot=0&num=20) | - | 1.75% | 0.865 | - | - | - |
| [Porphyromonadaceae](http://rdp.cme.msu.edu/seqmatch/seqmatch_sum.jsp?qvector=204&depth=0&currentRoot=0&num=20) | - | - | 0.86% | 0.73% | 5.32% | - |
| [Prevotellaceae](http://rdp.cme.msu.edu/seqmatch/seqmatch_sum.jsp?qvector=204&depth=0&currentRoot=0&num=20) | - | - | 0.86% | - | 14.79% | 63.04% |
| [Bifidobacteriaceae](http://rdp.cme.msu.edu/seqmatch/seqmatch_sum.jsp?qvector=204&depth=0&currentRoot=970&num=20) | - | - | - | - | 1.18% | - |
| [Victivallaceae](http://rdp.cme.msu.edu/seqmatch/seqmatch_sum.jsp?qvector=204&depth=0&currentRoot=0&num=20) | - | - | - | - | 1.18% | - |
| [Verrucomicrobiaceae](http://rdp.cme.msu.edu/seqmatch/seqmatch_sum.jsp?qvector=204&depth=0&currentRoot=0&num=20) | - | - | - | - | 1.18% | - |

(−) indicates no detection
