# Supplementary figures and images for: Changes in human gut flora with age: an Indian familial study
Source: BMC Microbiol. 2012 Sep 26;12:222. doi: 10.1186/1471-2180-12-222 (PMC3511239; doi:10.1186/1471-2180-12-222)

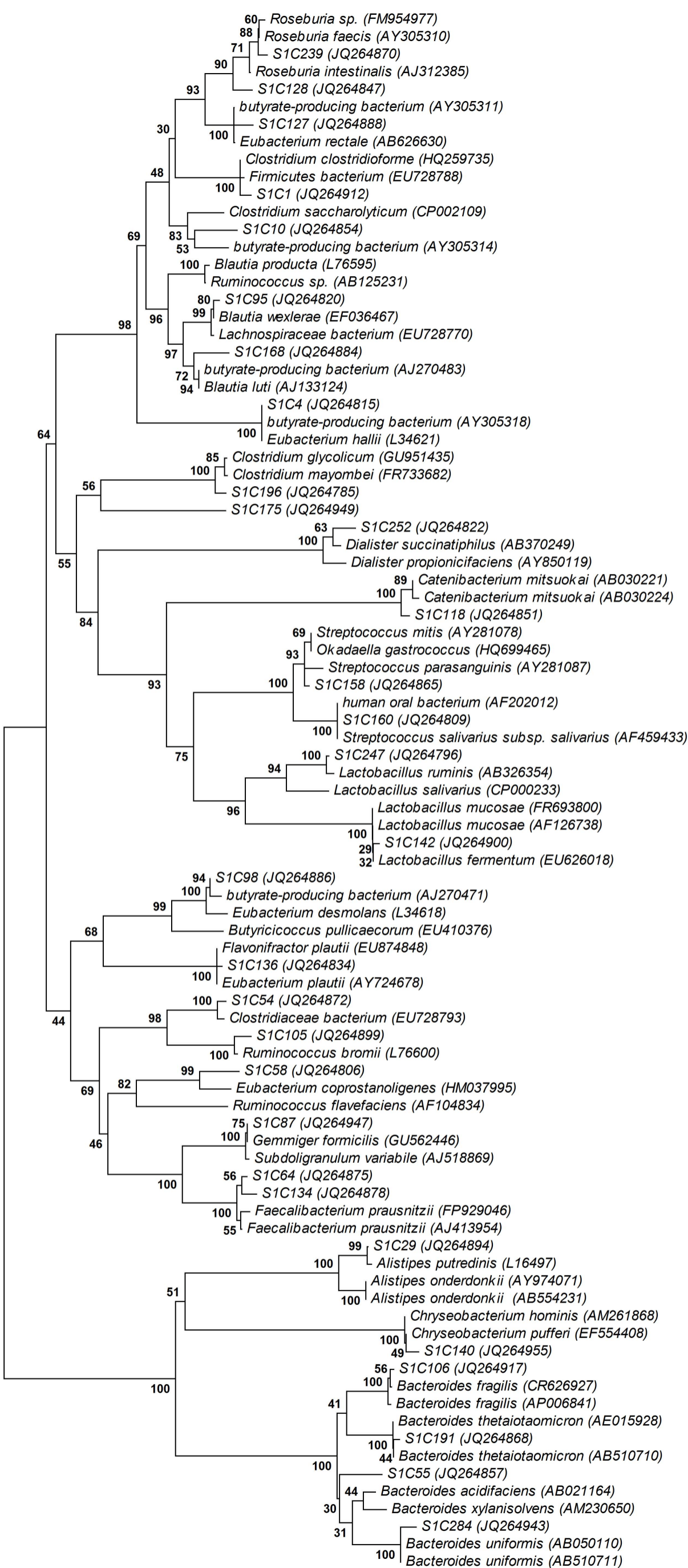

0.02

Supplement: Additional file 2 — Figure S1.Phylogenetic tree showing the position of 16S rDNA OTU’s recovered from stool sample of S1 individual was constructed using neighbor-joining method based on partial 16S rDNA sequences. The bootstrap values (expressed as percentages of 1000 replications) are shown at branch points. The scale bar represents genetic distance (2 substitutions per 100 nucleotides). GenBank accession numbers are in parentheses. [file 1471-2180-12-222-S2.pdf]

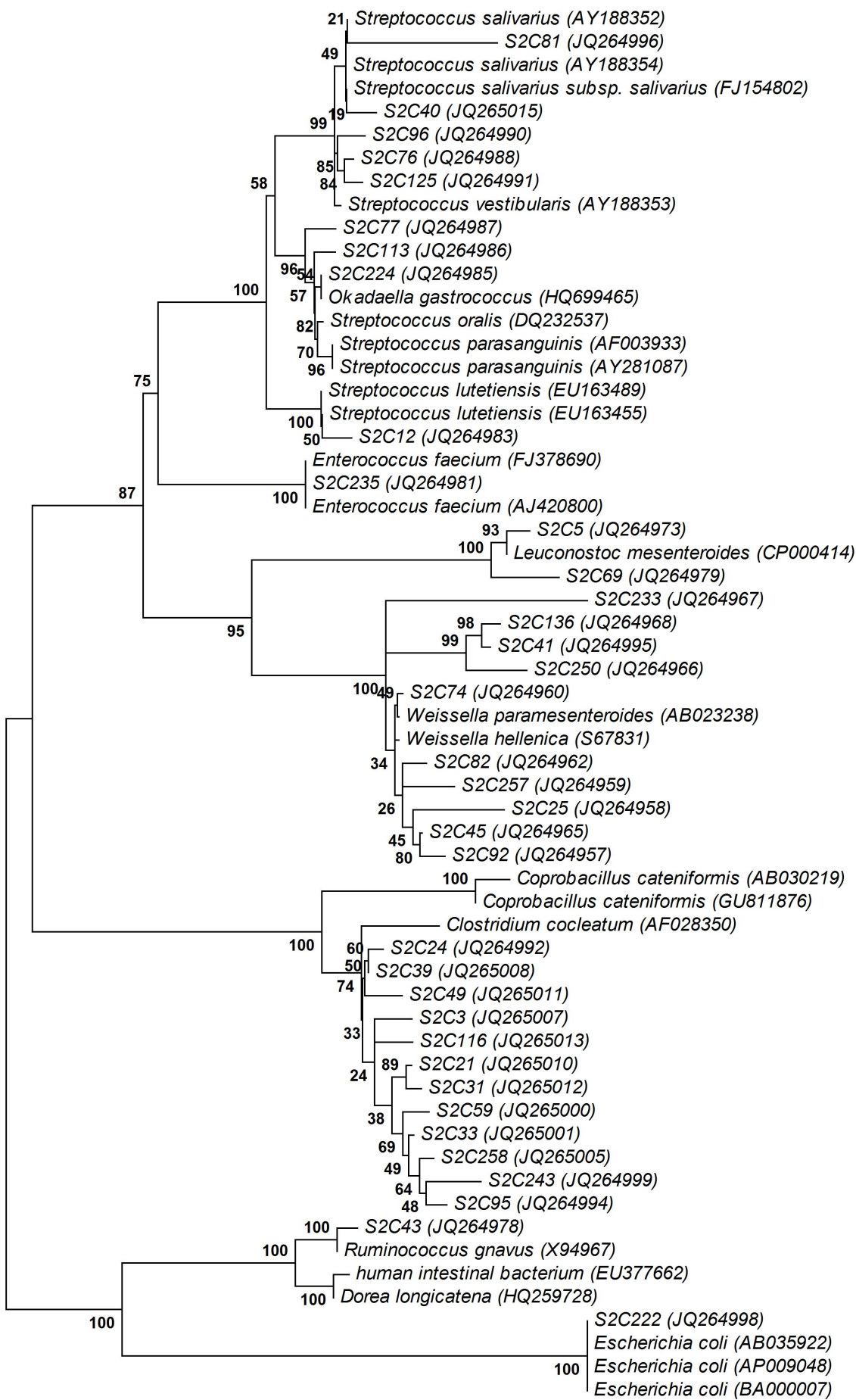

0.02

Supplement: Additional file 3 — Figure S2.Phylogenetic tree showing the position of 16S rDNA OTU’s recovered from stool sample of S2 individual was constructed using neighbor-joining method based on partial 16S rDNA sequences. The bootstrap values (expressed as percentages of 1000 replications) are shown at branch points. The scale bar represents genetic distance (2 substitutions per 100 nucleotides). GenBank accession numbers are in parentheses. [file 1471-2180-12-222-S3.pdf]

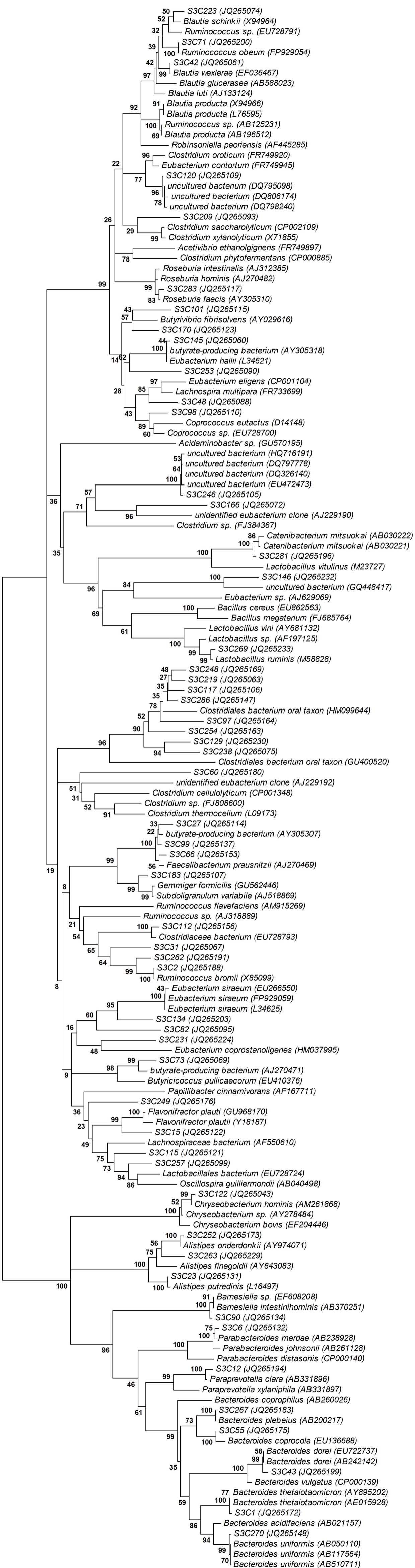

Supplement: Additional file 4 — Figure S3.Phylogenetic tree showing the position of 16S rDNA OTU’s recovered from stool sample of S3 individual was constructed using neighbor-joining method based on partial 16S rDNA sequences. The bootstrap values (expressed as percentages of 1000 replications) are shown at branch points. The scale bar represents genetic distance (2 substitutions per 100 nucleotides). GenBank accession numbers are in parentheses. [file 1471-2180-12-222-S4.pdf]

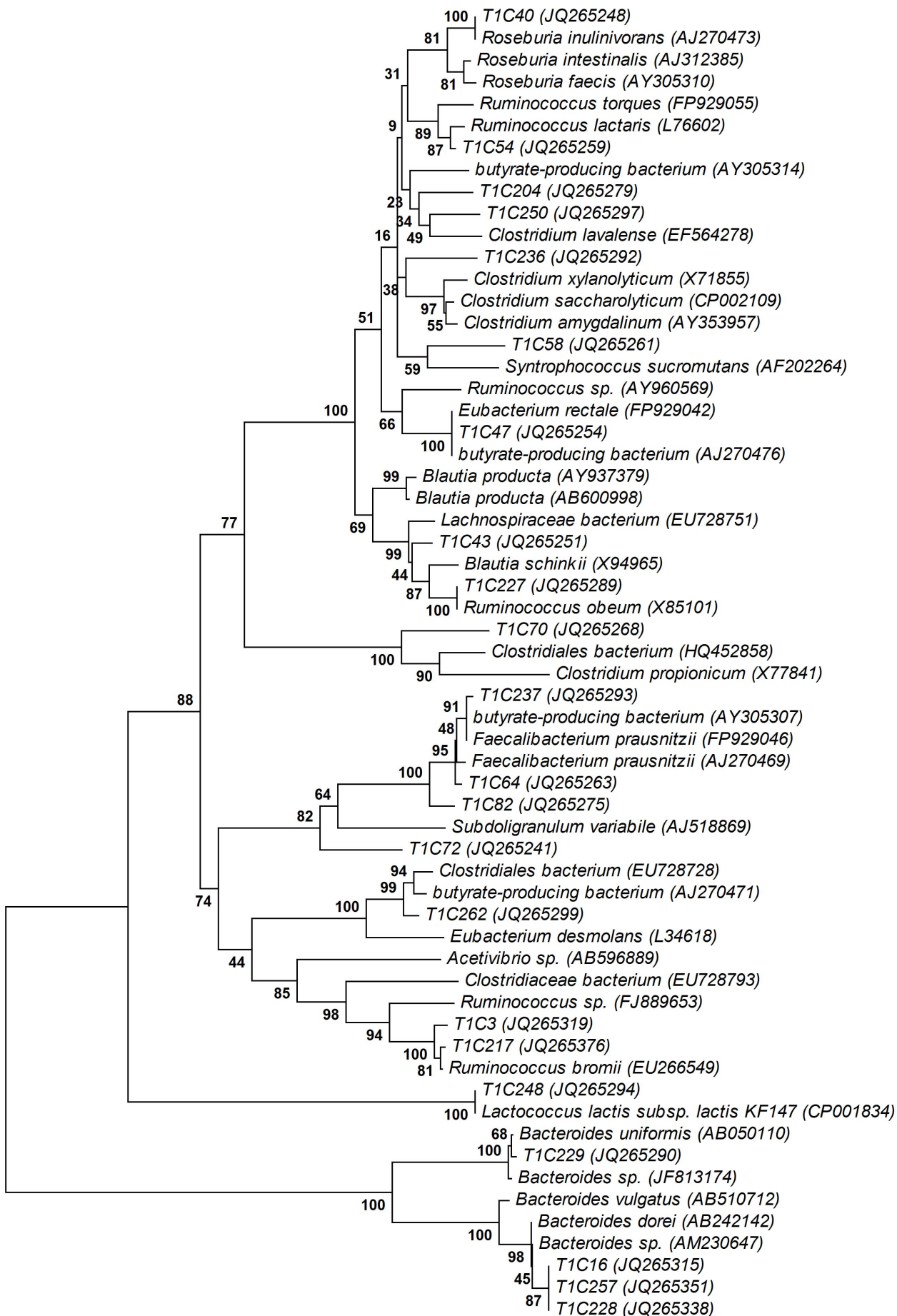

0.02

Supplement: Additional file 5 — Figure S4.Phylogenetic tree showing the position of 16S rDNA OTU’s recovered from stool sample of T1 individual was constructed using neighbor-joining method based on partial 16S rDNA sequences. The bootstrap values (expressed as percentages of 1000 replications) are shown at branch points. The scale bar represents genetic distance (2 substitutions per 100 nucleotides). GenBank accession numbers are in parentheses. [file 1471-2180-12-222-S5.pdf]

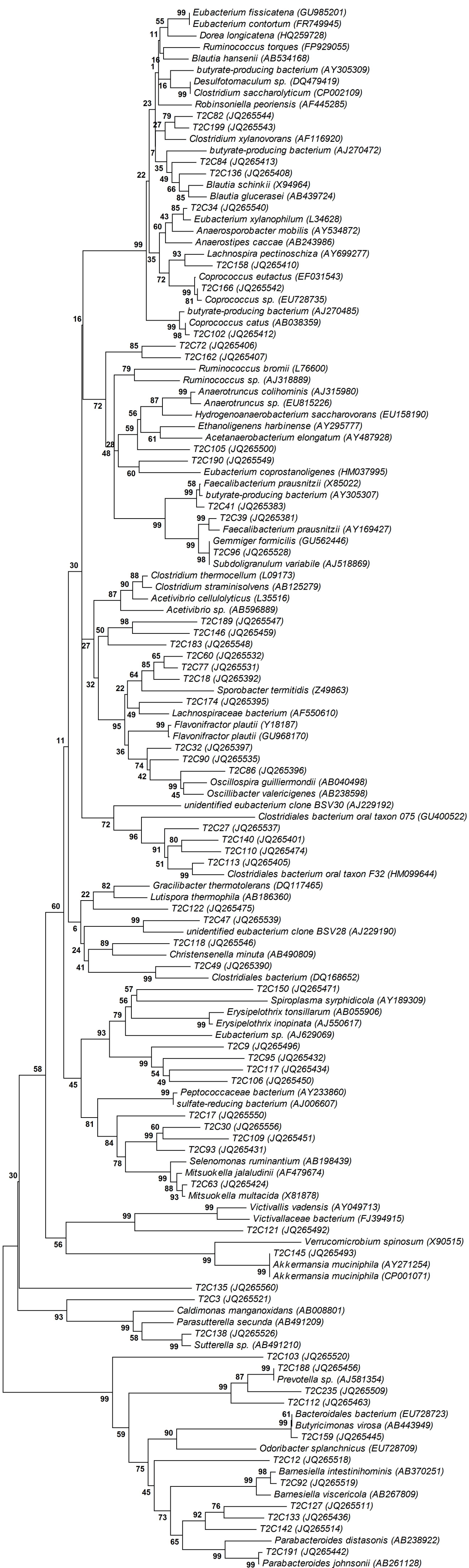

0.05

Supplement: Additional file 6 — Figure S5.Phylogenetic tree showing the position of 16S rDNA OTU’s recovered from stool sample of T2 individual was constructed using neighbor-joining method based on partial 16S rDNA sequences. The bootstrap values (expressed as percentages of 1000 replications) are shown at branch points. The scale bar represents genetic distance (5 substitutions per 100 nucleotides). GenBank accession numbers are in parentheses. [file 1471-2180-12-222-S6.pdf]

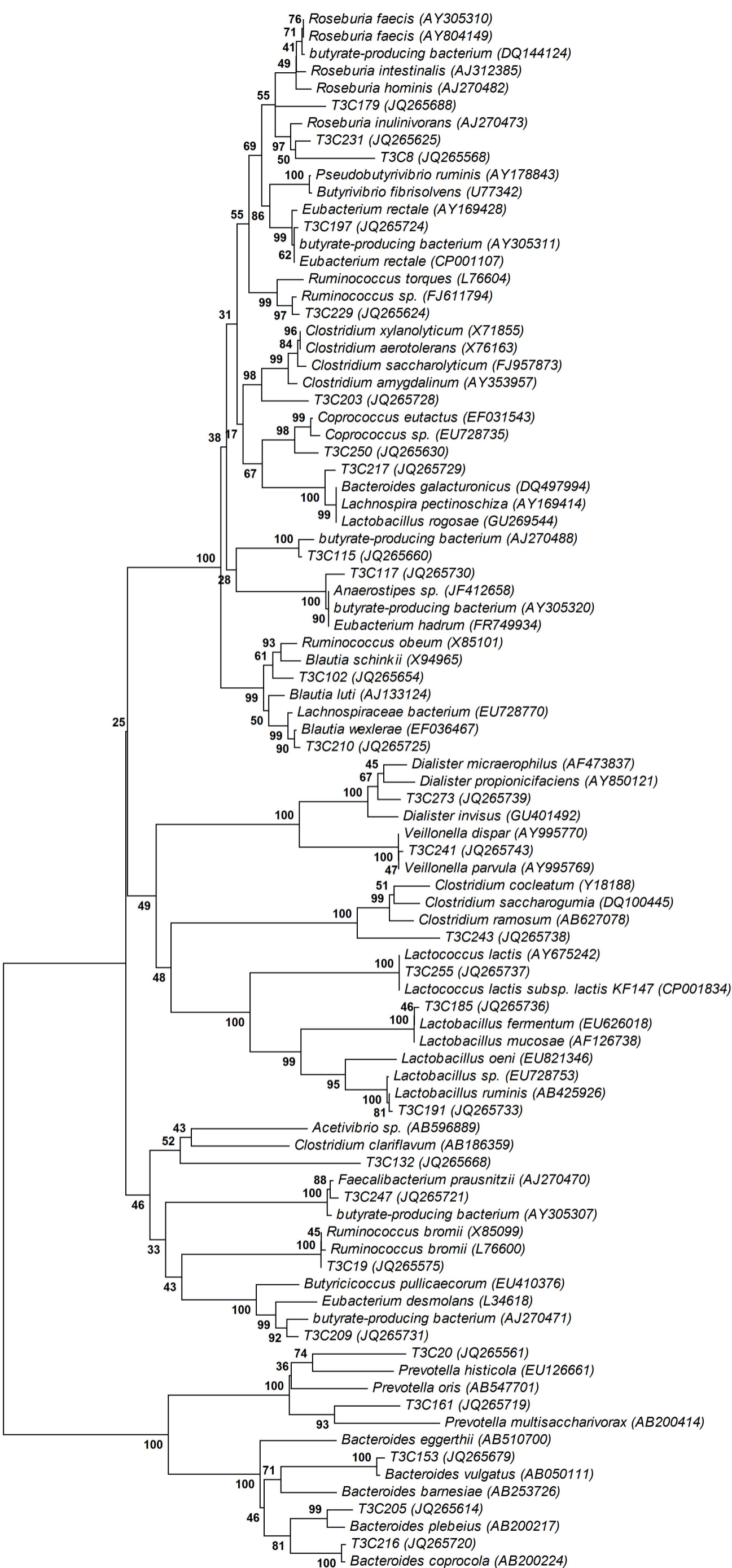

Supplement: Additional file 7 — Figure S6. Phylogenetic tree showing the position of 16S rDNA OTU’s recovered from stool sample of T3 individual was constructed using neighbor-joining method based on partial 16S rDNA sequences. The bootstrap values (expressed as percentages of 1000 replications) are shown at branch points. The scale bar represents genetic distance (5 substitutions per 100 nucleotides). GenBank accession numbers are in parentheses. [file 1471-2180-12-222-S7.pdf]
